# Research into Two Photonic-Integrated Waveguides Based on SiGe Material

**DOI:** 10.3390/ma13081877

**Published:** 2020-04-16

**Authors:** Song Feng, Bin Xue

**Affiliations:** School of Science, Xi’an Polytechnic University, Xi’an 710048, China; 20131005@xpu.edu.cn

**Keywords:** photonic-integrated, SiGe material, waveguide, losses

## Abstract

SiGe (Silicon Germanium) is a common semiconductor material with many excellent properties, and many photonic-integrated devices are designed and fabricated with SiGe material. In this paper, two photonic-integrated SiGe waveguides are researched, namely the SiGe-SOI (Silicon Germanium-Silicon-On-Insulator) waveguide and the SiGe-OI (Silicon Germanium-On-Insulator) waveguide. In order to verify which structure has the better waveguide performance, two waveguide structures are built, and the effective refractive indexes and the loss characteristics of the two waveguides are analyzed and compared. By simulation, the SiGe-OI optical waveguide has better losses characteristics at a wavelength of 1.55 μm. Finally, SiGe-OI and SiGe-SOI waveguides are fabricated and tested to verify the correctness of theoretical analysis, and the experimental results show that the transmission losses of the SiGe-OI waveguide are respectively decreased by 36.6% and 28.3% at 400 nm and 600 nm waveguide width in comparison with the SiGe-SOI waveguide. The results also show that the SiGe-OI waveguide has better loss characteristics than those of the SiGe-SOI waveguide at the low Ge content.

## 1. Introduction

The optical waveguide is the basic device of optical interconnection, and also is the basic unit of optical devices. With the progress of technology, the size of optical device is gradually reduced to the scale of 100 nanometers. Silicon on Insulator (SOI) material is widely used in the optical device field because SOI material has many advantages, such as good waveguide properties, and high compatibility, traditional microelectronic technology and mature processing technology, low cost, and so on. A waveguide with SOI material has the advantages of large refractive index difference, strong limiting effect for optical wave, low absorption loss, small device size, and the ability to increase the integration degree of the photonic circuit. Therefore, optical switches, modulators, filters and other optical devices with SOI waveguides can effectively realize optical interconnection, which is being researched by most research institutions at present [1,2]. 

SiGe is an alloy material which has many advantages for optoelectronic devices, such as high refractive index, high electron mobility, high compatibility, and SiGe material could be used in many optoelectronic devices [3,4,5]. The band gap [6] and refractive index [7] of Si_1-x_Ge_x_ alloy material could be changed by adjusting the Ge content. When the SiGe waveguide is implemented in SOI, the effective refractive index of the waveguide layer can be increased, which means that device sizes and losses can be decreased. The size of the waveguide also becomes smaller and smaller with decreasing feature size of the electronic device, and the cross-section dimension of the SiGe optical waveguide has been reached at a micro-nano level [8,9]. The development of the SiGe heterostructure devices has resulted in the SiGe/Si heterojunction optical waveguide having no doping, and its loss being very low [10]. With the development of SOI technology, SOI in optoelectronic integration is becoming more and more important, and the manufacture of most SiGe optical waveguides are currently based on SOI technology [11]. The SiGe optical waveguide technology based on the SOI substrate can effectively improve the dielectric refractive index of the waveguide, so as to further reduce the size of the device, reduce the loss and improve the integration. At present, most of the research on SiGe optical waveguides and optical devices focuses on small size SiGe-SOI waveguides, while there is little research on SiGe-OI optical waveguides and optical devices [12]. The main reason for this is that the growth technology of SiGe-OI material is under development, and most of the research is still focused on the growth of SiGe-OI materials. Therefore, there is good academic value and application prospects in developing SiGe-OI optical waveguide and optical devices by using SiGe optical waveguide technology.

SiGe waveguides can be used in communication band and the loss and size all are easy to control, so SiGe waveguide devices are the main research objects of many scientific research institutions. A single-mode rib SiGe waveguids is reported using the MBE-grown method, and the photodetectors made with this SiGe waveguide has a dark current of less than 200 nA at 7 V reverse bias. The quantum efficiency of the fiber-waveguide-detector is 11% overall at 7 V reverse bias, and a maximum bandwidth of 2 GHz has been achieved [13]. The Ge/SiGe quantum-well device made of SiGe material has the advantage of high ER, and low loss and energy consumption. The ER is 4 dB with an insertion loss of 3 dB, and the energy consumption is lower than 100 fJ/bit [14]. A silicon-based optical switch which has a carrierplasma-induced phase shifter is proposed using an SiGe/Si hetero-structure as the waveguide core. The fabricated Mach-Zehnder optical switch with SiGe material is fabricated. This has a low switching power of only 1.53 mW, and the compact phase shifter length is 250 μm. The switching time of the SiGe switch is less than 4.6 ns in a square waveform driving condition [15]. Si and Ge have different lattice sizes, so there are lattice stresses between Si and Ge. In order to reduce the lattice mismatch between Si and Ge, a new Ge-rich silicon-germanium waveguide is proposed with a graded buffer, which can be applied to the integration of germanium-rich active devices in photonic-integrated circuits. The extinction of Mach Zehnder interferometer with this Ge-rich silicon-germanium waveguide is higher than 10 dB, and the low loss bends with radii are 12 μm at a wavelength of 1550 nm [16]. The nonlinear signal processing system with the SiGe waveguide is suitable for high spectral efficiency data signals, and 64-QAM signals of this system is less than −10-dB conversion efficiency [17]. According to the plasma dispersion effect and the internal reflection, a SiGe/Si asymmetric optical waveguide switch is proposed at the wavelength of 1550 nm. The modulation depth of SiGe/Si waveguide switch is 90%, the switching time is about 0.2 ms, and the injection current is 110 mA. Its extinction ratio is more than 34 dB, and the insertion loss and the crosstalk are respectively less than 2.86 dB and 218.5 [18]. The nonlinear characterization of Ge-rich Si1-xGex waveguides is proposed, and its germanium concentrations range from 0.7 to 0.9 at a wavelength of 1.58 μm [19].

The SiGe waveguide can not only be used in communication band, but also be widely used in the research of middle infrared band with the increase of the research heat of middle infrared band devices in recent years [20,21,22]. A 2.5-cm long SiGe waveguide which is used in error-free transmission of 10-Gbit/s non-return to zero optical signals is grown by reduced pressure chemical vapor deposition (RP-CVD) at a wavelength of 1.98 μm. The thickness of SiGe waveguides is 1.4 μm with 20% germanium concentration (x = 0.2), and the width of SiGe waveguides is 1.3 μm and 2.2 μm respectively [23]. A Bragg-mirror based on SiGe waveguides is designed at a wavelength of 7.25 μm, which has a Q-factor around 104 at a low 1dB/cm propagation loss [24]. The characterization of SiGe/Si graded index waveguides and SiGe/Si photonics integrated devices are researched. SiGe/Si graded index waveguides cover the full 3–8 μm by optimizing the thickness and the Ge concentration of the SiGe material. The losses of straight SiGe waveguides are respectively lower 1 dB/cm at the wavelength of 4.5 μm and 2 dB/cm at the wavelength of 7.4 μm [10]. The Ge-rich SiGe alloy-based photonic structures are proposed for a wavelength range spanning from 5.5 to 8.5 μm [25]. The first third-order nonlinear experimental characterization of Ge-rich Si1-xGex waveguides is proposed, and its germanium concentrations x ranges from 0.7 to 0.9. The width of the Ge-rich Si1-xGex waveguide is 1.6 μm, and the thickness is 2 μm. The experimental results provide many helpful insights to assist the design of mid-infrared wavelength devices [26].

SiGe-SOI waveguide and SiGe-OI waveguide have two waveguide structures, which all can be fabricated with SOI epitaxial wafer. The fabricated structure of SiGe-SOI waveguide is such that the SiGe layer is grown directly on the SOI epitaxy substrate by molecular beam epitaxy [27] or chemical vapor deposition [28], and then the waveguide structure can be obtained by etching. For the SiGe-OI waveguide, SOI technology is used to fabricate SiGe-OI structure by SIMOX [29], bonding [30], Smart-cut [31], and Ge concentrate [32,33] technology, and then the waveguide structure can also be obtained by etching. Important questions include which one has more excellent characteristics, and how much Ge content is optimal. In this paper, the effective refractive indexes and loss characteristics of two photonic-integrated SiGe waveguides are analyzed, and Ge content of SiGe waveguide are optimized. Finally, SiGe-OI and SiGe-SOI waveguides are fabricated and tested, and the experimental results show that SiGe-OI waveguide has better loss characteristics than the SiGe-SOI waveguide.

## 2. Mechanism and Manufactures 

**Figure 1:**
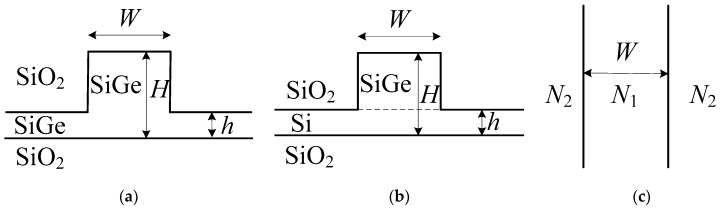
The structure of (**a**) Silicon Germanium-On-Insulator (SiGe-OI) optical waveguide, (**b**) Silicon Germanium-Silicon-On-Insulator (SiGe-SOI) optical waveguide and (**c**) equivalent model.

The model of SiGe waveguides are built by matrix elimination and the numerical method, and the structure of SiGe-OI and SiGe-SOI are respectively shown in Figure 1a,b. 

In Figure 1a,b, SiO_2_ is the substrate, SiGe and SiGe/Si are the waveguide layers, and the overlying layer is SiO_2_. H is the inner ridge height of waveguide, h is the outer ridge height, and W is the ridge width. According to planar effective index theory, the ridge waveguide structure can be equivalent to a one-dimensional planar structure, which is shown in Figure 1c. According to Maxwell theory, the effective refractive indexes *N*_1_ and *N*_2_ on the horizontal direction are calculated with matrix elimination (which is an algorithm in linear algebraic programming for solving linear systems of equations) and numerical method by the following Equation (1) [34]:

(1) $$N_{j}^{2}=n_{1}^{2}-\frac{{\left(m+1\right)}^{2}\pi^{2}}{{\left[k_{0}d_{j}+{\left(n_{1}^{2}-n_{2}^{2}\right)}^{-0.5}/\eta_{12}+{\left(n_{1}^{2}-n_{3}^{2}\right)}^{0.5}/\eta_{13}\right]}^{2}},\;j=1,\;2$$

where *N*_j_ is the effective refractive index at different vertical heights; *n*_1_ is the effective refractive index of waveguide, and *n*_1_ = 3.473 for Si material [6]; *n*_2_ and *n*_3_ are the effective refractive indexes of cladding layer, and *n*_2_ = *n*_3_ = 1.444 for SiO_2_ material; *η*_12_ and *η*_13_ is constant, and *η*_12_ = *η*_13_ = 1 for TE (Transverse-Electric) mode; *k*_0_ is the free-space wavenumber, and *k*_0_ = 2π/λ = 2π/1.55 at the wavelength of 1.55 μm; *d*_j_ is the height on the vertical direction for N*_j_* region; m is the order of guided mode.

**Figure 2:**
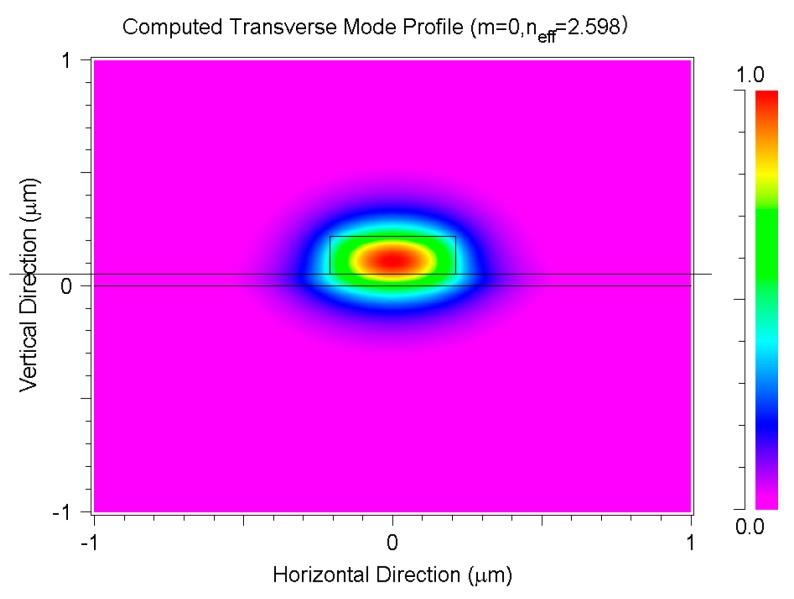
The fundamental TE-mode field and effective refractive index of SiGe-OI optical waveguide at the wavelength of 1.55 μm.

According to planar effective index theory, the final optimized parameters of waveguide structure are H = 220 nm, h = 50 nm, W = 400~600 nm [35]. In the structure of SiGe optical waveguide, SiO_2_ is used as the substrate whose refractive index is n_1_ = 1.444, and Si_1-x_Ge_x_ is used as the waveguide layer whose refractive index is n_2_ = 3.473 + 0.37x + 0.22x^2^ [6]. Si is used as the slab layer whose refractive index is n_3_ = 3.473 only in SiGe-SOI structure. At the wavelength of 1.55 μm the effective refractive indexes of SiGe-OI and SiGe-SOI are simulated by beam propagation method (BPM) [36], and effective refractive indexes of SiGe-OI and SiGe-SOI are 2.598 and 2.592, respectively. The *E*_x_ profile of SiGe-OI is shown in Figure 2, and the *E*_x_ profile of SiGe-SOI is almost the same as that of SiGe-OI. 

In order to verify the correctness of theoretical analysis, SiGe-OI and SiGe-SOI waveguides are fabricated. The structures of SiGe-OI waveguide and SiGe-SOI waveguide are adopted in Figure 1. 

For the production of the SiGe-SOI optical waveguide, we chose the silicon thickness of the top layer as 0.3 μm. SiGe-SOI optical waveguide devices were fabricated on 8-inch SOI chips with embedded silicon dioxide thickness of 3 μm. The main processes of producing SiGe-SOI optical waveguides are as follows:

1. Pretreatment of SOI substrate, mainly cleaning and dusting the surface of silicon wafer;
2. Thinning the SOI substrate;
3. Growing the SiGe layer on the top of the silicon membrane by UHV/CVD method;
4. Depositing the SiO_2_ layer by PECVD, and using the generated silica as the etching mask layer of SiGe;
5. Applying photoresist and using mask for lithography;
6. Putting the lithographic film into the developer for development, transfering the image from mask to photoresist and making the film hard;
7. Etching the silicon dioxide mask layer with photoresist as the mask, transferring the graphics from the photoresist layer to the silicon dioxide mask layer;
8. Using the silicon dioxide mask layer as the mask to etch the SiGe layer, transfering the graphics from the silicon dioxide mask layer to the SiGe layer, and completing the fabrication of the waveguide structure;
9. Removing photoresist, silicon dioxide mask layer and scratch.

Note: During the characteristic test, in order to protect the chip and also serve as the covering layer of waveguide, a SiO_2_ layer of PECVD should be deposited on the chip before the test. For the production of the SiGe-SOI optical waveguide, we purchased the SiGe-OI epitaxial wafer, and then made the waveguide by etching. The process is similar to that of SiGe-SOI.

The structure parameters of SiGe-OI waveguide and SiGe-SOI waveguide are shown in Table 1. 

**Figure 3:**
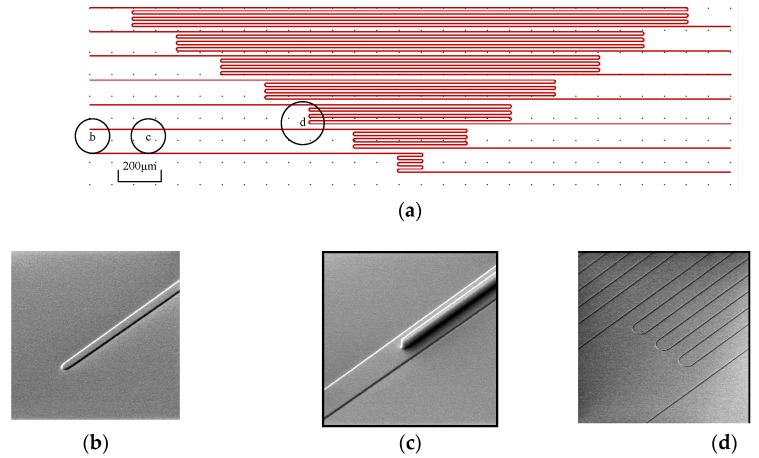
(**a**) SiGe waveguide layout, (**b**) tip waveguide, (**c**) the couple of SiGe waveguide and tip waveguide, (**d**) SiGe waveguide bending structure.

The SiGe-OI waveguide and SiGe-SOI waveguide are fabricated with existing process conditions. The layout and scanning electron microscope (SEM) photos of SiGe waveguide are shown in Figure 3.

The test instruments of optical waveguide mainly includes the ASE laser, tapered single-mode fiber, the polarization controller, optical spectrometer, and six dimensions of the platform. In the testing process of the optical waveguide, the waveguide is very small and there is no packaging. Moreover, the input/output optical fiber directly connected to the waveguide accuracy is not high, and the accuracy directly affects the device testing performance, so the optical alignment platform is needed for precise alignment. The optical alignment platform is used in testing, which can be adjusted in three axial directions and three angular directions. The adjustment accuracy of the optical alignment platform can reach 7 nm, which fully meets the accuracy requirements of testing.

Firstly, the tested chip is fixed on the objective table, and the input and output taper fiber are aligned with the waveguide by optical alignment platform. Then, the other end of the input optical fiber is connected to the ASE laser, and the output optical fiber is connected to the spectrometer or power meter. Finally, the light signal of the ASE laser light source using a taper fiber is connected to the chip, and the response spectrum can be seen on the spectrometer or the optical power can be seen on the power meter. 

## 3. Results and Discussion

**Figure 4:**
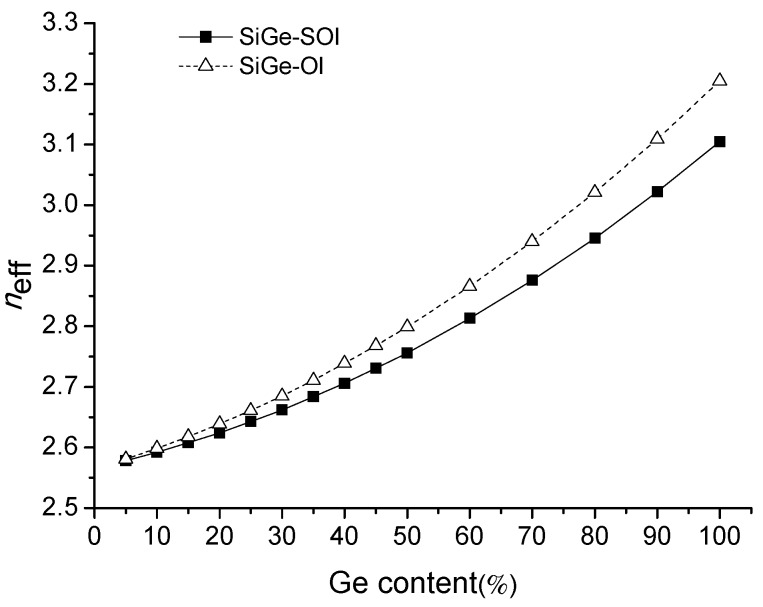
The relation curve between effective refractive index and Ge content at the wavelength of 1.55 μm.

**Figure 5:**
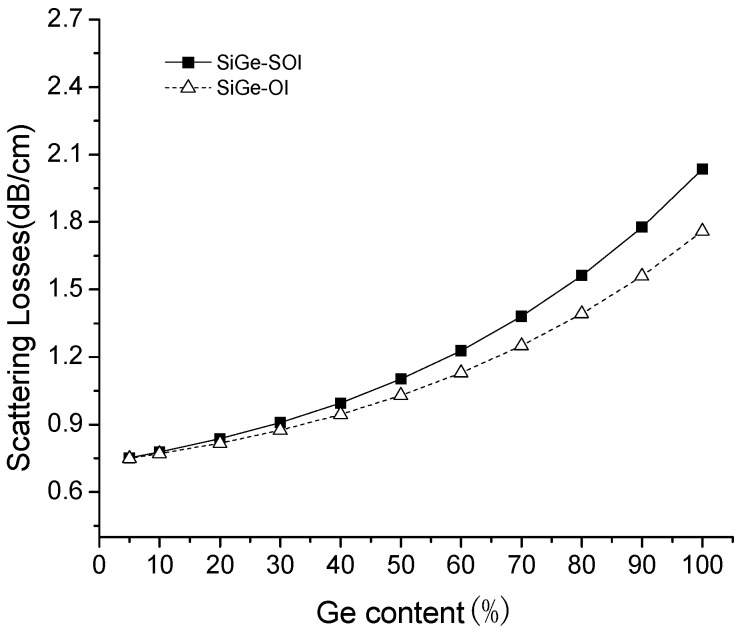
The relationship between scattering losses and Ge content at the wavelength of 1.55 μm.

**Figure 6:**
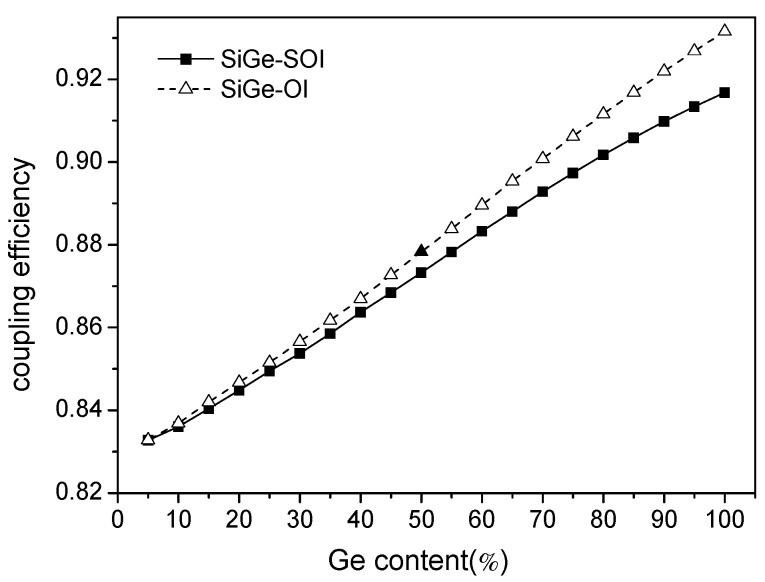
The relationship between coupling efficiency and Ge content at the wavelength of 1.55 μm.

**Figure 7:**
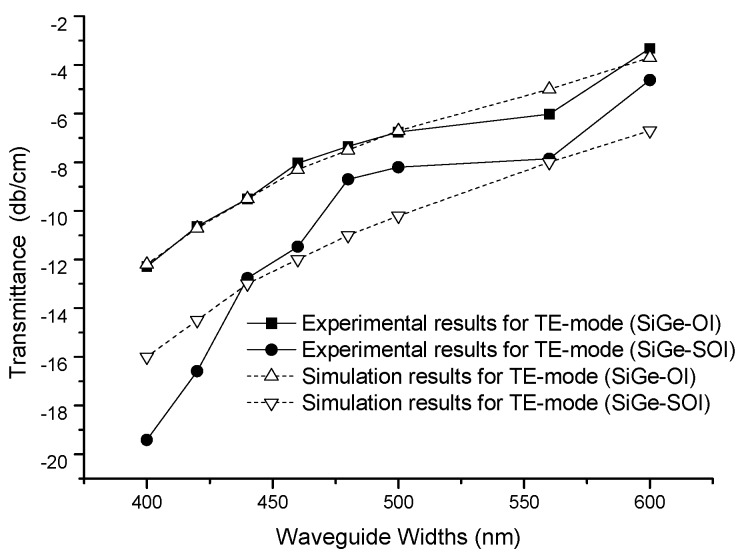
Experimental and simulation results of SiGe-OI and SiGe-SOI waveguide transmittance for TE-mode at the wavelength of 1.55 μm.

Figure 4, Figure 5 and Figure 6 below are shown as simulation results, and Figure 7 is a comparison of the test results and simulation results.

Ge content is an important parameter for SiGe optical waveguides, and the effective refractive indexes of SiGe waveguides can be changed by adjusting Ge content. The effective refractive indexes of two types of SiGe waveguides are shown in Figure 4 at different Ge content at the wavelength of 1.55 μm. From Figure 4, we can see that the effective refractive indexes of the two types of SiGe waveguides all increased with increased Ge content. The effective refractive indexes difference between the two waveguides can almost be ignored when the Ge content is low. With increased Ge content, the effective refractive index difference between two waveguides gradually increased. That is because the restriction capability of the SiGe slab layer for optical wave increases with increased Ge content.

The transmission losses of the waveguides include the absorption losses, leakage losses, and scattering losses. Interband absorption occurs when the photon energy is greater than the band gap width. At a wavelength of 1.55 μm, the photon energy is about 0.8 eV, which is less than the band gap width of SiGe material (when Ge content is 5%, the band gap width is 1.123 eV). Therefore, the absorption losses of SiGe can be neglected with small Ge content [37]. The optical wave is effectively restricted by the oxidation layer under slab layer, and leakage losses can be ignored [38]. In the manufacturing process of the waveguide, some defects, lattice damage and bubbles maybe appear on the interface between the core layer and the cladding layer of waveguide, or the roughness of waveguide is large in the etching process, which will cause the scattering losses of waveguide. Thus, the transmission losses of SiGe waveguide are mainly the scattering losses induced from side-wall roughness of the waveguide. The scattering losses of the SiGe-OI waveguide and SiGe-SOI waveguide are shown in Figure 5 with different Ge content at the wavelength of 1.55 μm, when absorption losses and leakage losses are ignored. In Figure 5, the scattering losses can be increased with increased Ge content. The scattering losses of the SiGe-SOI waveguide are higher than those of the SiGe-OI waveguide at the same Ge content. Moreover, the higher Ge content, the greater the difference in scattering losses between the SiGe-OI waveguide and SiGe-SOI waveguide. This is because the scattering losses can be increased with increased the side-wall roughness of the waveguide. Furthermore, the roughness can be increased with increased crystal defects such as stacking faults and dislocations in the SiGe epitaxial layer and SiGe/Si interface, and crystal defects can be increased with increased Ge content. The scattering losses in Figure 5 can be calculated according to Equation (2) [39,40,41]:

(2) $$\alpha_{s}=\frac{\sigma^{2}}{\sqrt{2}k_{0}d^{4}n_{1}}\cdot g(V)\cdot f_{e}(s,\gamma ),$$

where $g(V)=\frac{U^{2}V^{2}}{1+W}$; $f_{e}(s,\gamma )=\frac{s{\left\{{\left[{\left(1+s^{2}\right)}^{2}+2s^{2}\gamma^{2}\right]}^{0.5}+1-s^{2}\right\}}^{0.5}}{{\left[{\left(1+s^{2}\right)}^{2}+2s^{2}\gamma^{2}\right]}^{0.5}}$; $U=d\sqrt{k_{0}{}^{2}n_{1}{}^{2}-\beta^{2}}$; $V=k_{0}d\sqrt{n_{1}{}^{2}-n_{2}{}^{2}}$; $W=d\sqrt{\beta^{2}-k_{0}{}^{2}n_{2}{}^{2}}$; $s=W\frac{L_{c}}{d}$; $\gamma =\frac{n_{2}V}{n_{1}W}\sqrt{\Delta }$; $\Delta =\frac{n_{1}{}^{2}-n_{2}{}^{2}}{2n_{1}{}^{2}}$; *σ* the roughness of the waveguide, *k*_0_ is the free-space wavenumber, *d* is the half-width of the waveguide, *n*_1_ is the effective refractive index of the waveguide core region, *L*_c_ is the autocorrelation length, and *β* is the propagation constant. *σ* and *L*_c_ are very critical for the accurate calculation of scattering losses, and the scattering losses can be effectively decreased by reducing the roughness of the waveguide and autocorrelation length. *σ* = 2 nm and *L*_c_ = 50 nm in Figure 5.

Figure 6 is the coupling efficiency diagram of the ridge waveguide and fiber when Ge content changes from 0 to 1 at the wavelength of 1.55 μm. That can be seen in Figure 6. When Ge content is 0, the waveguide material is Si, and initial coupling efficiency is 82.88%. With the increase of Ge content, the effective refractive index becomes higher, the ridge waveguide has an increased ability to limit the field enhancement, and the SiGe optical waveguide and optical coupling efficiency increased gradually; thus, the coupling loss is reduced. When Ge content is at a fixed value, the coupling efficiency of SiGe-OI is greater than that of SiGe-SOI; when Ge content is low, the coupling efficiency of SiGe-OI and SiGe-SOI is almost the same; with the increase of Ge content, the coupling effect of SiGe-OI increases and is greater than that of SiGe-SOI.

The experimental and simulation results of the SiGe-OI and SiGe-SOI waveguide transmittance for TE-mode are shown in Figure 7 at the wavelength of 1.55 μm and the structure parameters of the SiGe-OI waveguide and SiGe-SOI waveguide are shown in Table 1. The simulation results of transmittance for SiGe-OI waveguide can be well matched with those of testing results. The simulation results of transmittance for the SiGe-SOI waveguide are different from those of testing results at some waveguide widths. This may be due to the influence of the roughness of waveguide interface in the manufacturing process or material growth. From Figure 7 we can see that transmittance of the SiGe-OI waveguide and SiGe-SOI waveguide all are decreased with increased waveguide width. When the waveguide width is 400 nm, the testing values of transmittance for SiGe-OI and SiGe-SOI waveguides are respectively −12.3 dB/cm and −19.4 dB/cm, and the simulation value of transmittance for SiGe-OI and SiGe-SOI waveguides are respectively −12.2 dB/cm and −16 dB/cm. The testing and simulation values of transmittance for the SiGe-OI waveguide are decreased by 36.6% and 23.8% in comparison with the SiGe-SOI waveguide. When the waveguide width is 600 nm, the testing values of transmittance for the SiGe-OI and SiGe-SOI waveguides are respectively −3.3 dB/cm and −4.6 dB/cm, and the simulation values of transmittance for the SiGe-OI and SiGe-SOI waveguides are respectively −3.7 dB/cm and −6.7 dB/cm. The testing and simulation values of transmittance for the SiGe-OI waveguide are decreased by 28.3% and 44.8% in comparison with the SiGe-SOI waveguide. So, from that we can see, the SiGe-OI waveguide has better transmission characteristics.

The composition of transmission loss includes leakage loss, scattering loss and absorption loss. The effect of absorption loss and leakage loss of conventional optical waveguide on the device is very small and can almost be ignored [37,38]. Therefore, only the effect of scattering loss caused by side wall roughness in waveguide etching on the device needs to be considered. However, when the size of the SiGe optical waveguide device is reduced, the strain SiGe layer will also bring about an increase in scattering loss. Since there is stress between Si atoms and Ge atoms in the strain SiGe layer during the growth process, there is strain energy in the strain SiGe layer, which will make the surface of the device rough. Moreover, the Ge content is higher, the strain energy is larger and the surface roughness is higher. When the section size of the ridged waveguide is large, the waveguide has a strong limiting ability to the light field. The light field is basically confined in the core layer, and the scattering loss occurring on the side wall is small. When the section size of the ridged waveguide is less than one transmission wavelength, the light field has a strong distribution at the boundary of the waveguide core layer, and the light wave has a large scattering loss on the side wall of the waveguide, which results in a large discrepancy between the test results and the simulation results. With the reduction of SiGe optical waveguide size, the addition of Ge content has a great influence on the roughness of the waveguide’s side wall, so that the scattering loss of the waveguide increases. The above analysis is our speculation based on the experimental data for this time according to the related references [39,40,41]. SiGe waveguide is likely to be used for some active devices. For example, the SiGe waveguide can be applied to a micro-nano Si/SiGe/Si double heterojunction electro-optic modulation structure, which can greatly improve the carrier injection concentration, and decrease modulation voltage and power [42].

## 4. Conclusions

In conclusion, two photonic-integrated SiGe waveguide structures are obtainable with SOI technology. One is the SiGe-SOI optical waveguide, and the other is the SiGe-OI optical waveguide. Based on theoretical analysis, the preferred structural parameters are H = 220 nm, h = 50 nm, W = 400~600 nm, and the effective refractive indexes and loss characteristics are analyzed for the SiGe-SOI waveguide and the SiGe-OI waveguide. The effective refractive indexes of the two SiGe waveguides all increased with increased Ge content, and effective refractive indexes of SiGe-OI waveguide are higher than those of the SiGe-OI waveguide. The scattering losses of the two SiGe waveguides also increased with increased Ge content, and the scattering losses of the SiGe-OI waveguide are lower than those of the SiGe-SOI waveguide at low Ge contents. SiGe-OI and SiGe-SOI waveguides are fabricated and tested, and the final results show that transmission losses of the SiGe-OI waveguide are respectively decreased by 36.6% and 28.3% at 400 nm and 600 nm waveguide width in comparison with those of the SiGe-SOI waveguide. Thus, the losses of the SiGe-OI waveguide are lower than those of the SiGe-SOI waveguide at low Ge content.

## Figures and Tables

**Table 1:** Structure parameters of SiGe-OI waveguide and SiGe-SOI waveguide.

Ge	*H*/nm	*h*/nm	*W*/nm
5%	220	50	400
			420
			440
			460
			480
			500
			560
			600
